# Two-Dimensional TiS_2_ Nanosheet- and Conjugated Polymer Nanoparticle-Based Composites for Sensing Applications

**DOI:** 10.1021/acs.langmuir.4c03102

**Published:** 2024-10-15

**Authors:** D. Yeniterzi, S. C. Cevher, S. Kandur Baglicakoglu, A. D. Ucar, M. B. Durukan, T. Haciefendioglu, E. Yildirim, A. Cirpan, H. E. Unalan, S. Soylemez

**Affiliations:** † Department of Biomedical Engineering, Necmettin Erbakan University, 42090 Konya, Türkiye; ‡ Science and Technology Research and Application Center (BİTAM), Necmettin Erbakan University, 42090 Konya, Türkiye; § Department of Chemistry, Middle East Technical University, 06800 Ankara, Türkiye; ∥ Department of Metallurgical and Materials Engineering, Middle East Technical University (METU), 06800 Ankara, Türkiye; ⊥ Energy Storage Materials and Devices Research Center (ENDAM), Middle East Technical University (METU), 06800 Ankara, Türkiye

## Abstract

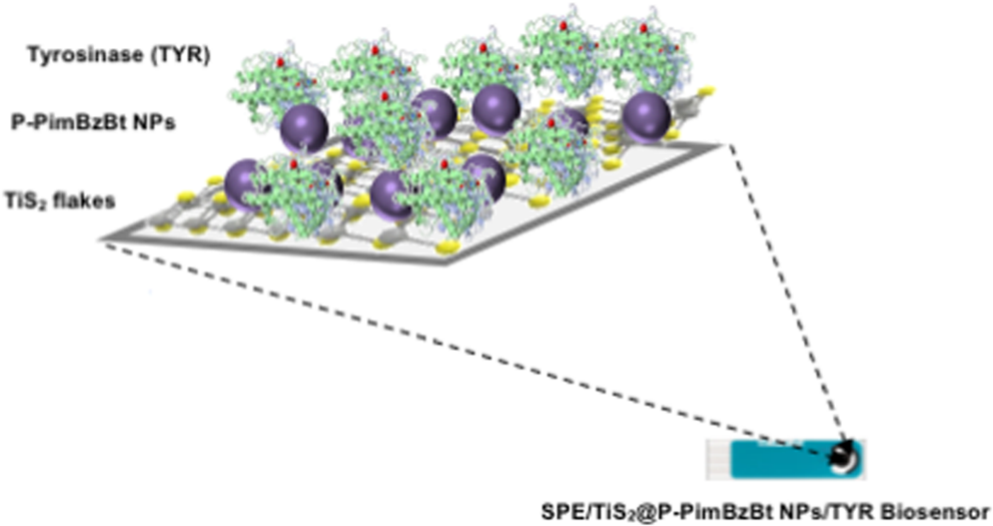

The assessment of phenolic compounds in food samples, environmental samples, and medical applications has gained importance recently. Here, we present research on novel conjugated polymer nanoparticles (P-PimBzBt NPs) and their composites with two-dimensional titanium disulfide nanosheets (2D-TiS_2_) for electrochemical tyrosinase (TYR)-based catechol detection. P-PimBzBt NPs are decorated with 2D-TiS_2_ to enhance the electrochemical performance for biosensing. In addition, the interaction of P-PimBzBt NPs with TiS_2_ was investigated at the molecular level by employing van der Waals (vdW) dispersion-corrected density functional theory (DFT) calculations and classical all-atom molecular dynamics simulations. According to the theoretical studies, the presence of the TiS_2_ layer increases the interfacial interaction with the conjugated polymer via electrostatic interactions. Using cyclic voltammetry (CV), electrochemical impedance spectroscopy (EIS), field emission scanning electron microscopy (FE-SEM), and energy-dispersive X-ray spectroscopy (EDS) analyses, the production of SPE/TiS_2_@P-PimBzBt NPs/TYR nanobiosensors was examined. With a detection range of 3.0–27.5 μM, 0.33 μM LOD, and 3.89 μA/μM·cm^2^ sensitivity values, the sensing layer based on the TiS_2_@P-PimBzBt NP composites has a targeting ability toward catechol. Its selectivity was investigated using commonly used interfering ions and compounds such as citric acid, urea, glucose, uric acid, KCl, and NaCl. Application of nanobiosensors to actual samples (tap water and black tea) was carried out with high accuracy. The fabricated biosensing platform demonstrates that P-PimBzBt NPs with 2D-TiS_2_ nanomaterial functionalization are appropriate as electrode materials and could be used to create an inexpensive, fast-response, and highly selective electrochemical biosensor for the detection of catechol in actual samples.

## 1 Introduction

A class of materials, known as conjugated polymers (CPs), possessing alternating single and multiple bonds along the backbone structure, enable efficient delocalization of electrons or holes throughout the polymer backbone. The distinctive electronic structure of CPs contributes to a variety of intriguing properties, such as semiconducting behavior, optical activity, and electro/electrochemical activity. As a result, they are utilized in various fields for a wide range of applications.^1−5^ The remarkable electronic and optical properties, along with their processability and potential for cost-effective manufacturing, have led to widespread interest in CPs across multiple domains, such as electronics, optoelectronics, and energy storage. The field of biosensors, among others, is particularly suited to using CPs. Polymers used in biosensors offer signal amplification, versatility in design, and integration into portable devices for real-time monitoring. With their minimal cytotoxicity and long-term stability, CPs serve as essential tools in medical diagnostics and environmental monitoring.^6^ In addition, the benzotriazole core is a versatile acceptor moiety for the conjugated polymer backbone to tune optic and electronic properties. The availability of an alkyl chain on the triazole group enables the resulting conjugated polymer solution to be processable. On the other hand, bithiophene and its derivatives,^7,8^ within the subgroup family of thiophene, are prominent donor core structures for organic electronic materials. A combination of those donor and acceptor moieties produces CPs with distinct optic, electronic, and optoelectronic properties.^9^ On the other hand, the nanoparticles that can be produced from conjugated polymers exhibit numerous advantages and outstanding properties. A simple and straightforward preparation in tunable size, shape, and properties enables them to be great candidates for various applications such as sensing bioimaging, photodynamic and photothermal therapy, and bacteria killing.^10−13^ Significantly, the combination of electrochemical properties with photophysical features in a confined space, such as nanoparticles and conjugated polymer nanoparticles (CPNs), is a choice of material for advanced studies.

**Scheme 1:**
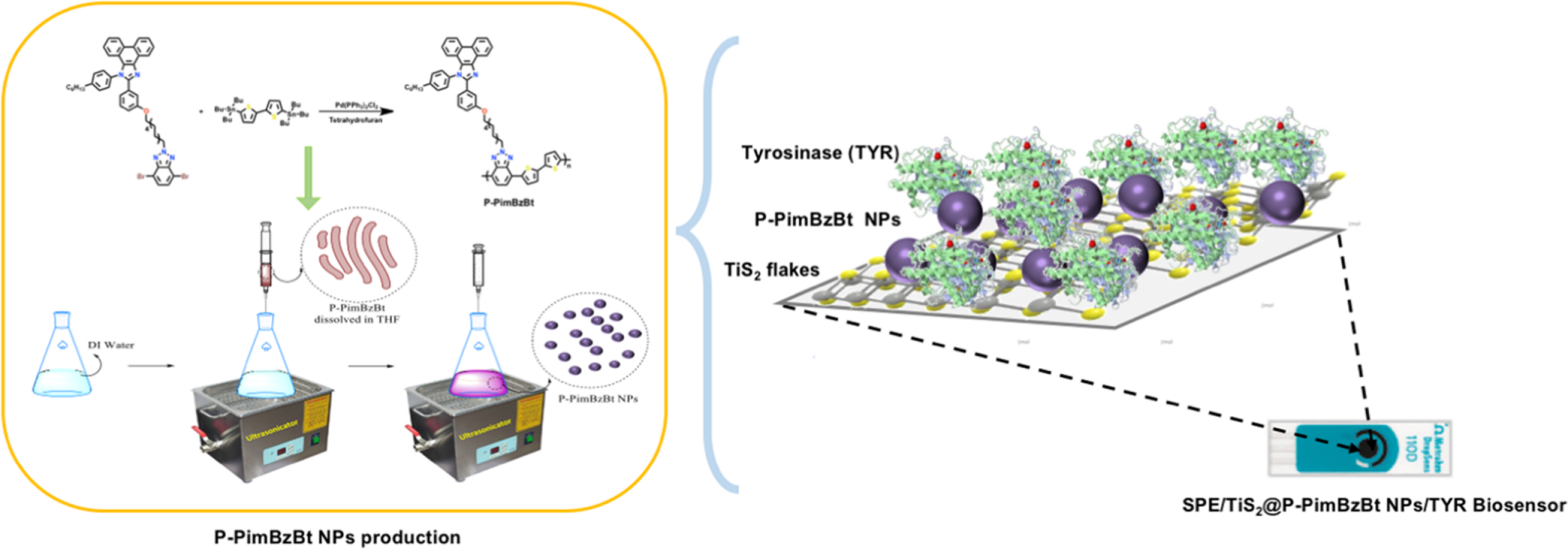
Cartoon Illustration of the SPE/TiS_2_/P-PimBzBt NPs/TYR Electrochemical Biosensor for Catechol Detection

Titanium disulfide (TiS_2_) is the lightest member of transition metal dichalcogenides (TMDs).^14,15^ Like other TMDs, it has the general formula of MX_2_ (M = Ti, X = S) and the titanium atom is positioned in a sandwich structure between two sulfur atoms, forming a S–Ti–S layer.^16^ Two-dimensional (2D) TiS_2_ can be obtained through the breaking of weak van der Waals bonds between layers. Its unique properties have made TiS_2_ suitable for use in batteries, photonics, and thermoelectric and biomedical applications.^16−21^ Furthermore, catalytic properties of TiS_2_ have been investigated and it has been shown to be a promising material.^22−25^ However, little attention has been paid to the synthesis and applications of the TiS_2_ nanosheet-based architecture for biosensor applications.^26^ For example, in a study^27^ where the supercritical exfoliation method was used to obtain a composite of molybdenum disulfide (MoS_2_) and poly(3-hexylthiophene) (P3HT), electrochemical properties of MoS_2_ could be tuned according to the ratios of MoS_2_ and P3HT. Another study^28^ revealed the differentiation of adsorbed and desorbed rhodamine dye in terms of fluorescence to detect the silver content in a living cell. The common feature of those is the surface interaction of the MXene-type materials with conjugated structures. Moreover, due to the crystal structure and the metal–chalcogen bond formation, TiS_2_ possesses more active surface edges than MoS_2_ for the adsorption and electrocatalytic oxidation of processes.^29^ Bearing these facts in mind, we synthesized a composite electrode material (TiS_2_:P-PimBzBt NPs) that exhibited excellent electrochemical performances via a synergistic effect to establish a highly capable electrochemical sensor. In addition, an electrochemical sensor for directly detecting catechol based on the TiS_2_:P-PimBzBt NP composite was constructed for catechol sensing. To the best of our knowledge, the effect of the combination of 2D-TiS_2_ and conjugated polymer nanoparticles on biosensor responses was evaluated for the first time. TiS_2_:P-PimBzBt NP-functionalized TYR was used for selective detection of catechol by an electrochemical method, as shown in Scheme 1.

## 2 Materials and Methods

### 2.1 Reagents and Apparatus

Tetrahydrofuran (THF) used to make conjugated polymeric nanoparticles was purchased from Honeywell (Inhibitor-free, Germany). Synthesis of the polymer P-PimBzBt was previously published.^30^ Titanium (99%, Nanografi), sulfur (99.98%, Sigma-Aldrich) and *n*-butyl lithium (1.6 M in hexane, Sigma-Aldrich) were used for the fabrication of bulk and 2D TiS_2_. For the purpose of making phosphate buffer solution (PBS), the salts sodium hydroxide (NaOH) and sodium phosphate dibasic (Na_2_HPO_4_·2H_2_O) were acquired from Merck (KGaA Darmstadt, Germany). Mushroom tyrosinase (25 KU, lyophilized powder, the activity of 1712 units per mg of solid), pyrocatechol (roughly 99%), glutaraldehyde (50 wt % in H_2_O) as the cross-linking agent, and sodium phosphate monobasic (NaH_2_PO_4_) were acquired from Sigma-Aldrich (St. Louis, MO).

Electrochemical studies were carried out using the AUTOLAB PGSTAT 204 analysis system, supported by a NOVA software package (Metrohm AG, The Netherlands) using Metrohm Dropsens C110 screen-printed electrodes with 4 mm diameter as the working electrode. Three repetitions of amperometric readings were made, and the averages were reported. All of the measurements were done at room temperature.

Furthermore, for the characterization of synthesized conjugated polymers, a Bruker Spectrospin Avance DPX-400 spectrometer with tetramethylsilane (TMS) as the internal reference was used to record nuclear magnetic resonance (NMR) spectra. Gel permeation chromatography (GPC) was performed on a Shimadzu RID-20A calibrated against polystyrene standards with a chloroform solvent to investigate the molecular weight. A Pyris 1 thermogravimetric analyzer (TGA) and a PerkinElmer Diamond differential scanning calorimeter (DSC) were used to investigate the thermal properties of the polymers. Using the FEI Quanta 400F model, field emission scanning electron microscopy (FE-SEM) images were taken of the prepared electrodes. For the characterization of synthesized conjugated nanoparticles, a dynamic light scattering (DLS) spectrometer (MALVERN/DLS MPT2), a fluorescence spectrophotometer (Agilent Cary Eclipse), a UV–vis–NIR spectrophotometer (Shimadzu UV-3600 Plus), and a high-contrast transmission electron microscope (HC-TEM) (FEI Tecnai G2 Spirit Biotwin) were used.

Morphological characterization of TiS_2_ structures was made via FE-SEM and a FEI Nova Nano FEG-SEM. X-ray diffraction (XRD) analyses were made using a Rigaku D/Max-2000 diffractometer employing Cu Kα radiation at an operating voltage of 40 kV. HC-TEM images of TiS_2_ were taken via a JEOL JEM-2100F with a 200 kV operating voltage.

### 2.2 Synthesis of the P-PimBzBt Polymer

**Figure 1:**
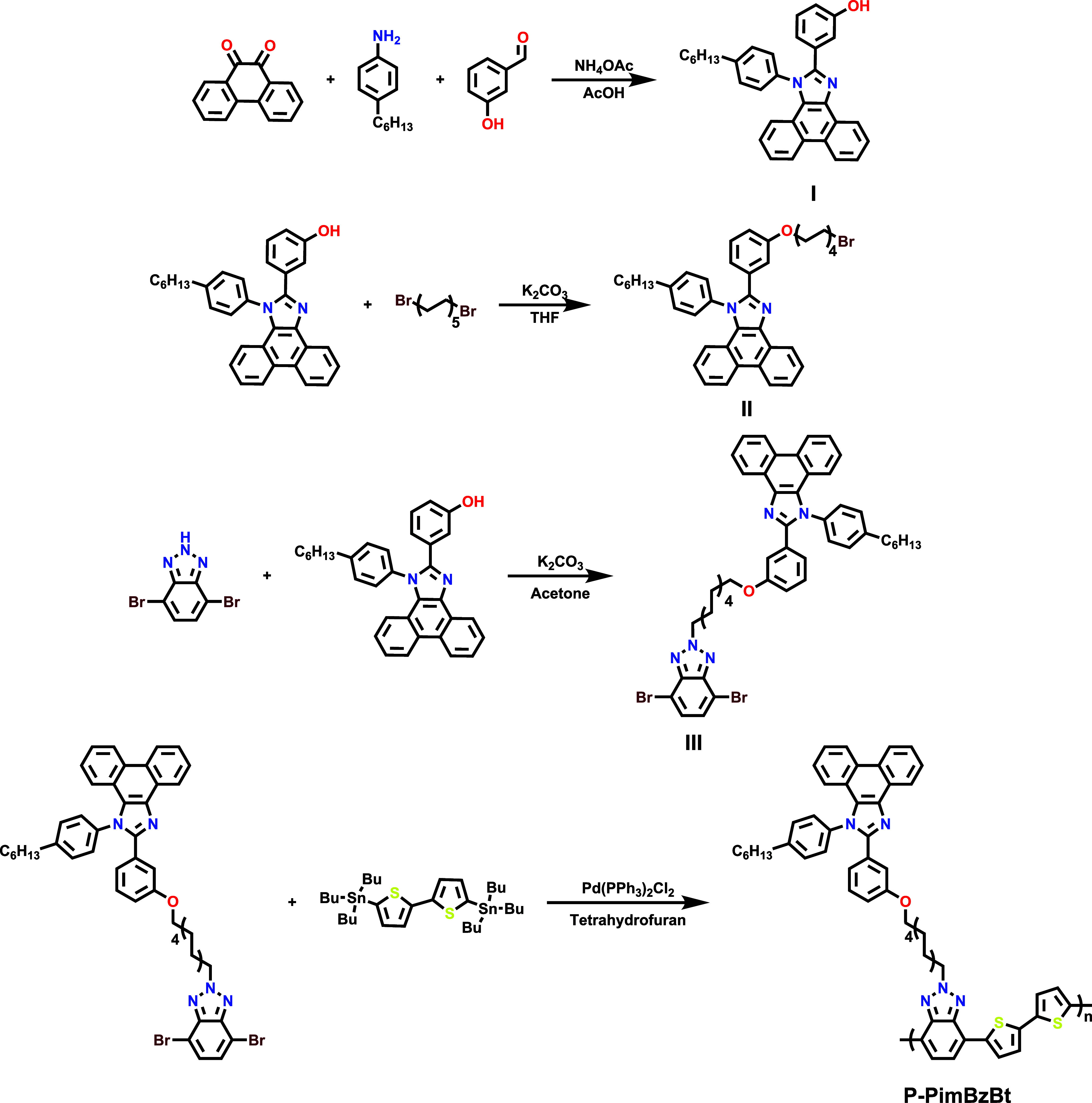
Synthetic pathway of P-PimBzBt.

Synthesis of the polymer P-PimBzBt was previously published, and it was performed as follows.^30^ The phenanthroimidazole core structure was built up by the four-component (9,10-phenanthrenequinone, 3-hydroxybenzaldehyde, 4-hexylaniline, and ammonium acetate, respectively) reaction in acetic acid at reflux. After completion of the reaction, the acetic acid mixture was cooled down and poured into the ice–water mixture. The obtained white solid was washed with excessive distilled water, and then the solids were dissolved in acetone and precipitated (compound **I**) by the addition of water. Then, compound **II** was dissolved in acetone, and dibromodecane and K_2_CO_3_ were added to the mixture and refluxed. After completion of the reaction, acetone was removed under reduced pressure, and extraction was performed with chloroform and water. The organic portion was collected, and the solvent was removed under reduced pressure, and column chromatography (chloroform) was used to purify product **III**. Later on, product **III** was attached to the 2-position triazole cycle on 4,7-dibromo-1*H*-benzo[*d*][1,2,3]triazole. Here, product **III** and the benzotriazole derivative were dissolved in tetrahydrofuran, K_2_CO_3_ was added, and the reaction was completed at reflux. Then, the solvent was removed, and extraction was performed with chloroform and water. The organic portion was collected, and the solvent was removed under reduced pressure. To purify the obtained monomer, column chromatography (chloroform-hexane) was performed. Later, the synthesized monomer and commercially available 5,5′-bis(tributylstannyl)-2,2′-bithiophene were reacted via a palladium-catalyzed cross-coupling reaction under a N_2_ atmosphere in THF at reflux. After the polymerization reaction, the concentrated polymer solution was precipitated into methanol, and the obtained solid was purified by Soxhlet extraction in the following order: methanol, acetone, and hexane. Finally, Soxhlet extraction with the chloroform polymer was collected and reprecipitated into methanol to obtain the polymer P-PimBzBt in a solid form (Figure 1). GPC: *M*_n_ = 4.8 kDa, *M*_w_ = 13.5 kDa, PDI = 2.8. ^1^H NMR: (400 MHz, CDCl_3_) δ 8.79 (s, 1H), 8.60 (s, 2H), 7.95 (s, 1H), 7.56 (s, 6H), 7.29 (s, 5H), 7.06 (s, 5H), 6.77–6.73 (m, 2H), 4.77 (s, 2H), 3.70 (s, 2H), 2.65 (s, 2H), 2.10 (s, 2H), 1.60 (s, 12H), 1.35–1.1 (m, 38H), 0.81 (s, 9H). TGA: *T*_decomposition_ 95% at 350 °C. DSC: No distinct thermal behavior was observed between 20 and 250 °C.

### 2.3 Synthesis of P-PimBzBt NPs

The P-PimBzBt NPs were prepared via the well-established reprecipitation method.^31^ 1 mg of P-PimBzBt was weighed and added to 5 mL of THF. It was left to dissolve in an ultrasonic bath for 3–4 min. The P-PimBzBt polymer was allowed to dissolve well in THF. It was withdrawn from the prepared stock polymer solution with a 1 mL syringe. It was injected into a glass bottle containing 20 mL of ultrapure water while in an ultrasonic bath. It was kept in an ultrasonic bath for 1 min. Then, it was left open in the fume hood for 24 h to allow the THF solution to evaporate. After 24 h, the solution was filtered through a 0.45 μM polystyrene syringe filter. As a result, P-PimBzBt nanoparticles ready for analysis were obtained.

### 2.4 Synthesis of 2D-TiS_2_

Stoichiometrically mixed powders of titanium and sulfur were annealed in a vacuumed quartz tube at 700° for 24 h to obtain bulk TiS_2_. After 48 hours of stirring with 20 mL of *n*-butyllithium (1.6 M in hexane) in a glovebox under argon atmosphere, lithium-intercalated TiS_2_ (Li-TiS_2_) was obtained through centrifuging with hexane, ethanol, and deionized water at 10,000 rpm for 30 min. Li-TiS_2_ powders were sonicated in deionized water for 3 h for delamination to obtain 2D-TiS_2_. 2D-TiS_2_ flakes were collected through centrifugation and stored in ethanol solution for further use.

### 2.5 Computational Methods

Two types of modeling calculations were performed for graphene@P-PimBzBt and graphene/TiS_2_@P-PimBzBt complexes at the molecular level.

**Figure 2:**
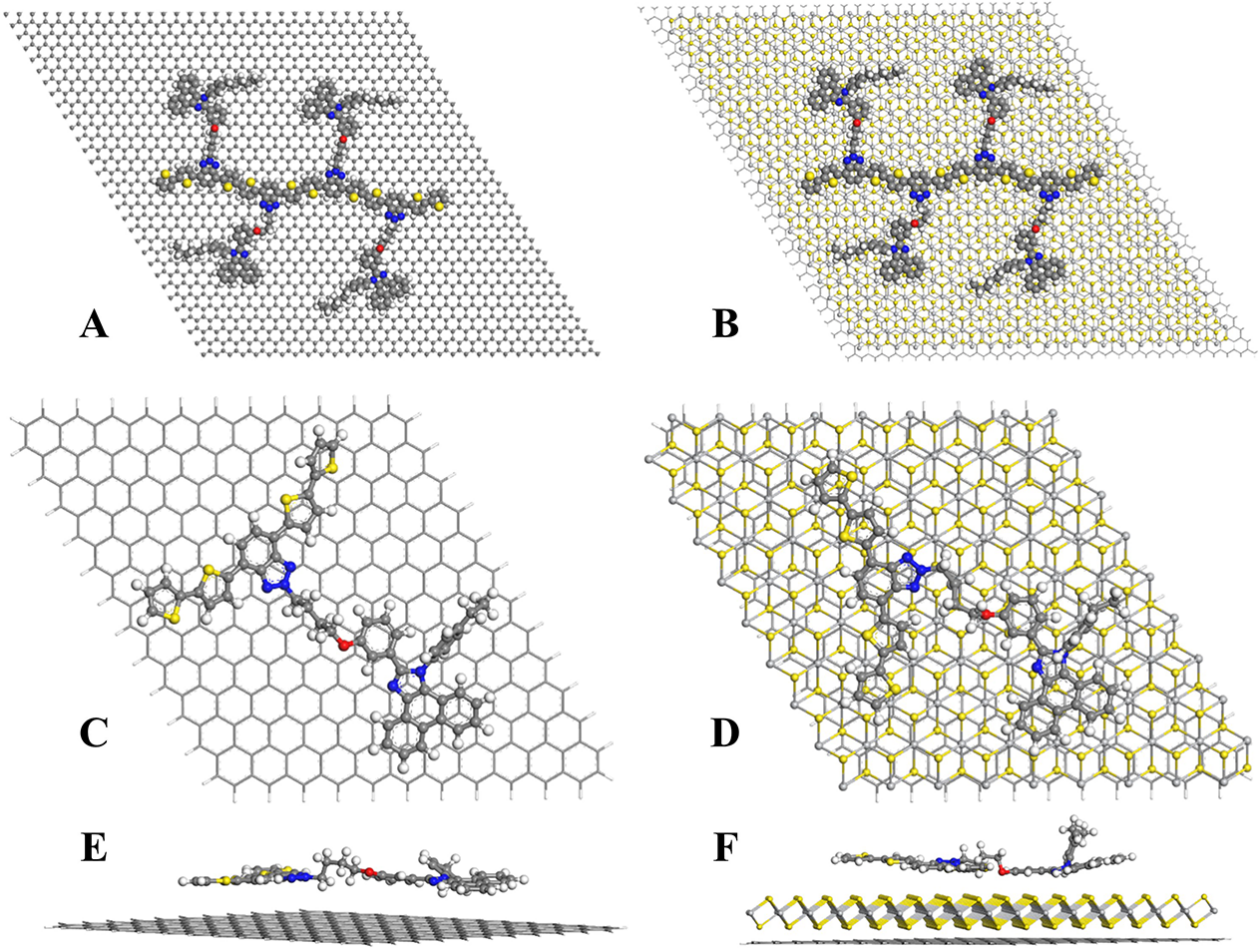
Initial structures of tetramers on (A) graphene@P-PimBzBt and (B) graphene/TiS_2_@P-PimBzBt surfaces for MD simulations. Initial structures of aromatic repeat units on (C–E) graphene@P-PimBzBt and (D–F) graphene/TiS_2_@P-PimBzBt surfaces for first-principle π calculations.

In the first calculations, the tetramer oligomer of the P-PimBzBt structure was studied by all-atom molecular dynamics (MD) simulations of graphene and graphene/TiS_2_ (Figure 2A,B). The class-II SciPCFF force field was used for the calculations in the simulations, which is a Scienomics MAPS^32^ version of the polymer consistent force field (PCFF).^33^ Atomic charges were calculated by using the Qeq charge equilibration method.^34^ Simulations were performed for 2 ns by 1 fs steps using the NVT ensemble under periodic boundary conditions in the LAMMPS simulation software.^35^

In the second calculation, single-point DFT calculations were performed for the repeating unit of the P-PimBzBt chains on graphene and graphene/TiS_2_. The structures were optimized by semiempirical methods to determine the position of the repeat unit on the surface where alkyl side chains and alkyl chain extensions were ignored to avoid a high computational expense for the system with 700 hundred atoms for graphene/TiS_2_ (Figure 2C–F). The Perdew–Burke–Ernzerhof (PBE) functional with the vdW dispersion correction was used in the calculations to calculate charge transfer on the surface.^36^

Graphene and TiS_2_ layers were fixed during the optimization of the P-PimBzBt position on the surface.

### 2.6 Preparation of the SPE/TiS_2_@P-PimBzBt NPs/TYR Nanobiosensor

The surface of the SPEs was cleaned with distilled water and then dried under N_2_ before each electrode modification. For electrode modification, as a first step, 1 μL of the 2D TiS_2_ nanomaterial suspension (diluted as a 1:5 ratio) was dropped onto the working surface of the SPE and allowed to dry at room temperature. Then, 6 μL of P-PimBzBt NPs were dropped onto the SPE/TiS_2_ surface and allowed to dry at room temperature in the next step. In the final step, 3 μL of the TYR enzyme was dropped on the surface of SPE/TiS_2_@P-PimBzBt NPs. TYR was immediately immobilized by cross-linking the SPE/TiS_2_@P-PimBzBt NPs/TYR surface by adding 3 μL of 1% GA cross-linking agent. The prepared and dried nanobiosensors were kept at +4 °C in a refrigerator until measurement.

### 2.7 Catechol Detection Using the SPE/TiS_2_@P-PimBzBt NPs/TYR Nanobiosensor

Chronoamperometry was used to analyze the SPE/TiS_2_@P-PimBzBt NPs/TYR biosensor. The experiments were carried out at a constant voltage of −200 mV in 10 mL of a 50 mM pH 7.0 phosphate buffer solution with gentle stirring. Steady-state amperometric currents were measured before and after catechol application to record a biosensor response. TYR catalyzes the oxidation of catechol to *o*-quinone in the presence of molecular oxygen followed by the electrochemical reduction of liberated *o*-quinone species.^37,38^

### 2.8 Preparation of Real Samples

The black tea sample was prepared according to the procedure in the literature.^39^ The quantification of catechol in each extract solution was determined in 10 mL of 0.1 M phosphate buffer solution by adding catechol solution at different concentrations. On the contrary, tap water samples were used directly without any pretreatment.

## 3 Results and Discussion

### 3.1 Characterization of TiS_2_ and P-PimBzBt PNPs

**Figure 3:**
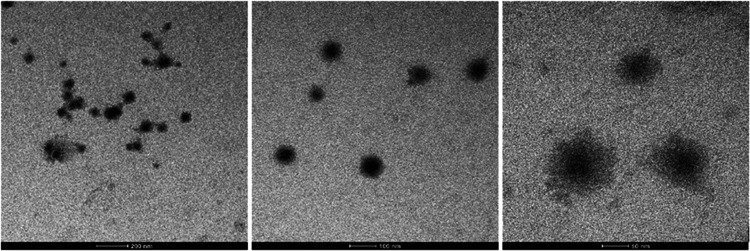
TEM images of synthesized P-PimBzBt NPs.

**Figure 4:**
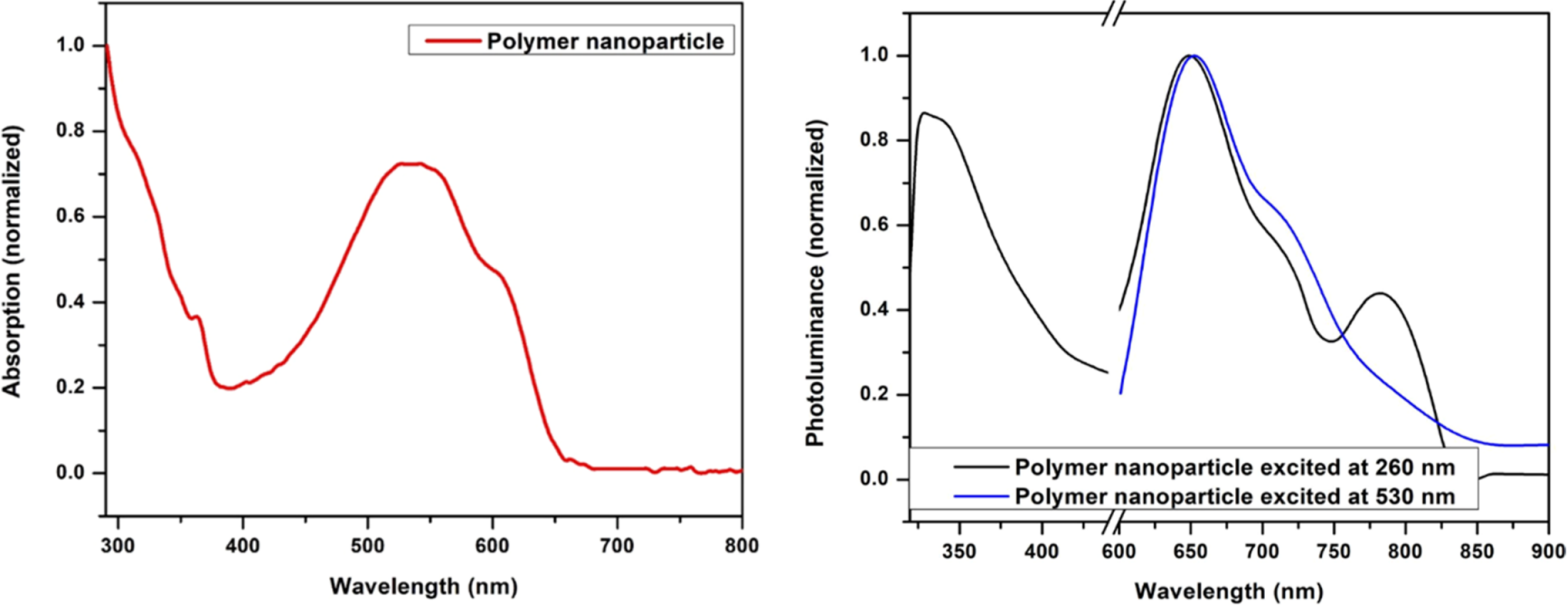
Photoluminance of the conjugated nanoparticles.

The TEM images of the produced P-PimBzBt PNPs demonstrate that their morphology is a uniform, monodisperse spherical shape (Figure 3). In the size distribution histogram that we presented to support the TEM analysis, it was observed that the hydrodynamic radius of the produced nanoparticles was 94 nm on average (Figure 3). DLS results of the P-PimBzBt PNPs are given in Figure S1. Furthermore, Figure 3 displays surface charge measurements of the produced nanoparticles acquired by a ζ potential analysis. This outcome shows that the produced nanoparticles have a surface charge of −60.52 mV (Figure S2). Similar to a previous study,^30^ P-PimBzBt PNPs exhibited a distinct photoluminance curve when excited at a short wavelength. Excitation at 256 nm gave both short- and long-wavelength dual emissions resulting from imidazole and the conjugated polymer chain, respectively. The conjugated polymer backbone fluorescence feature resembled the same excitation of either the polymer backbone or the imidazole. The peak around 800 nm might be attributed to the strong interaction of the conjugated polymer backbone in the confined space of the nanoparticle (Figure 4).

**Figure 5:**
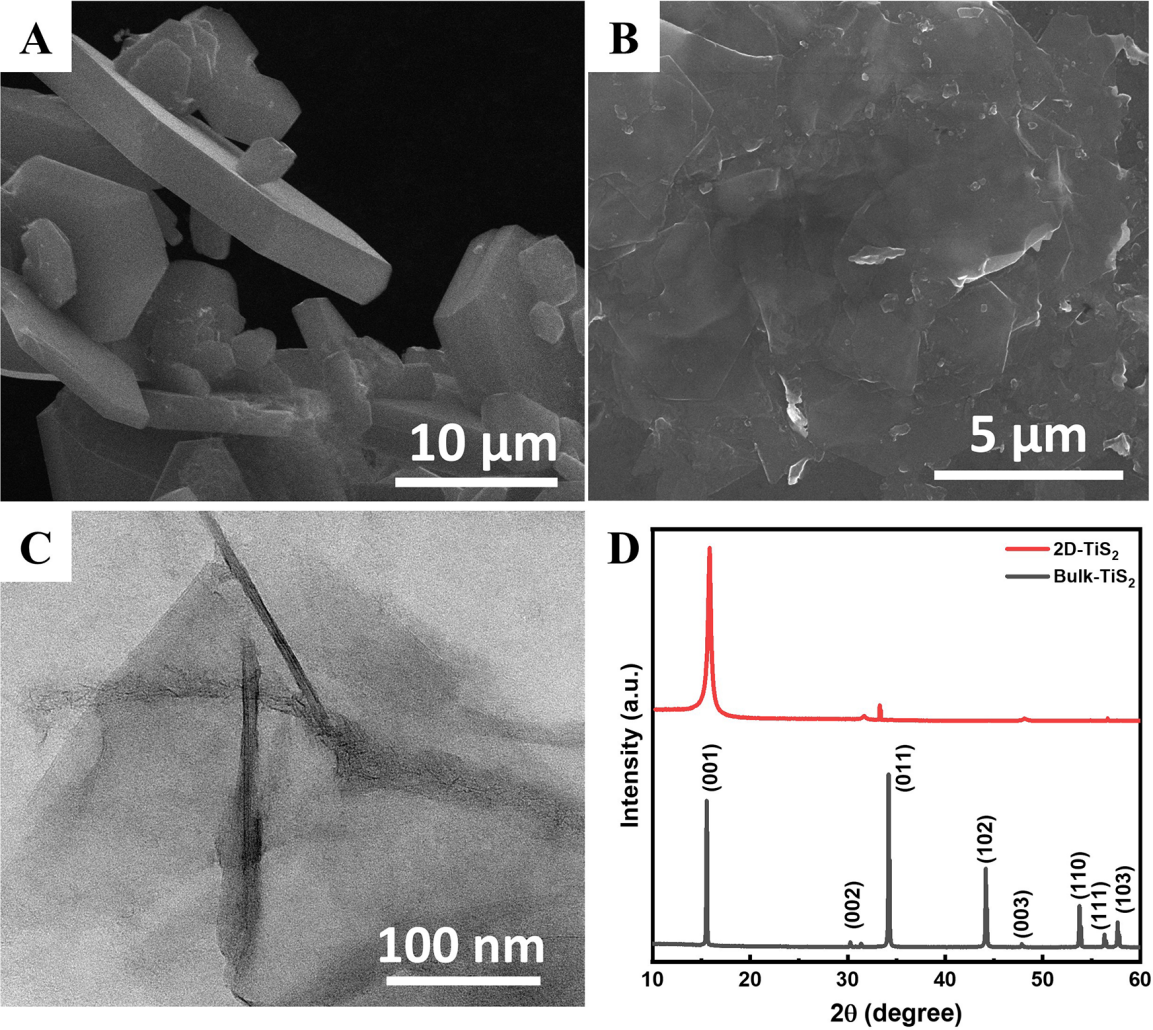
SEM images of (A) bulk TiS_2_ and (B) 2D-TiS_2_ nanosheets. (C) HC-TEM images of TiS_2_ nanosheets. (D) XRD patterns of bulk TiS_2_ and 2D-TiS_2_ nanosheets.

Detailed material characterization for exfoliated TiS_2_ is provided in Figure 5. Exfoliation of bulk TiS_2_ (Figure 5A) using *n*-butyllithium yields nanosheets of TiS_2_ (Figure 5B). The formation of nanosheets was further proven through HC-TEM imaging, which showed few-layered nanosheets (Figure 5C). XRD analysis was also done to prove the exfoliation chemically. Diffraction patterns of both bulk and 2D-TiS_2_ are given in Figure 5D. Diffraction of bulk TiS_2_ powders was consistent with the typical hexagonal formation of TiS_2_ (JCPDS card no. 88-1967). Upon exfoliation, broadening in the (001) peak was observed, while the remainder of the diffraction peaks disappeared. This is due to the exfoliation of the hexagonal structure of TiS_2_, leaving 2D nanosheets with mostly a characteristic diffraction of (001). This further proved the fabrication of a few-layered 2D-TiS_2_ through an organolithium exfoliation procedure.

### 3.2 Electrochemical and Surface Characterizations of the Prepared Electrodes

**Figure 6:**
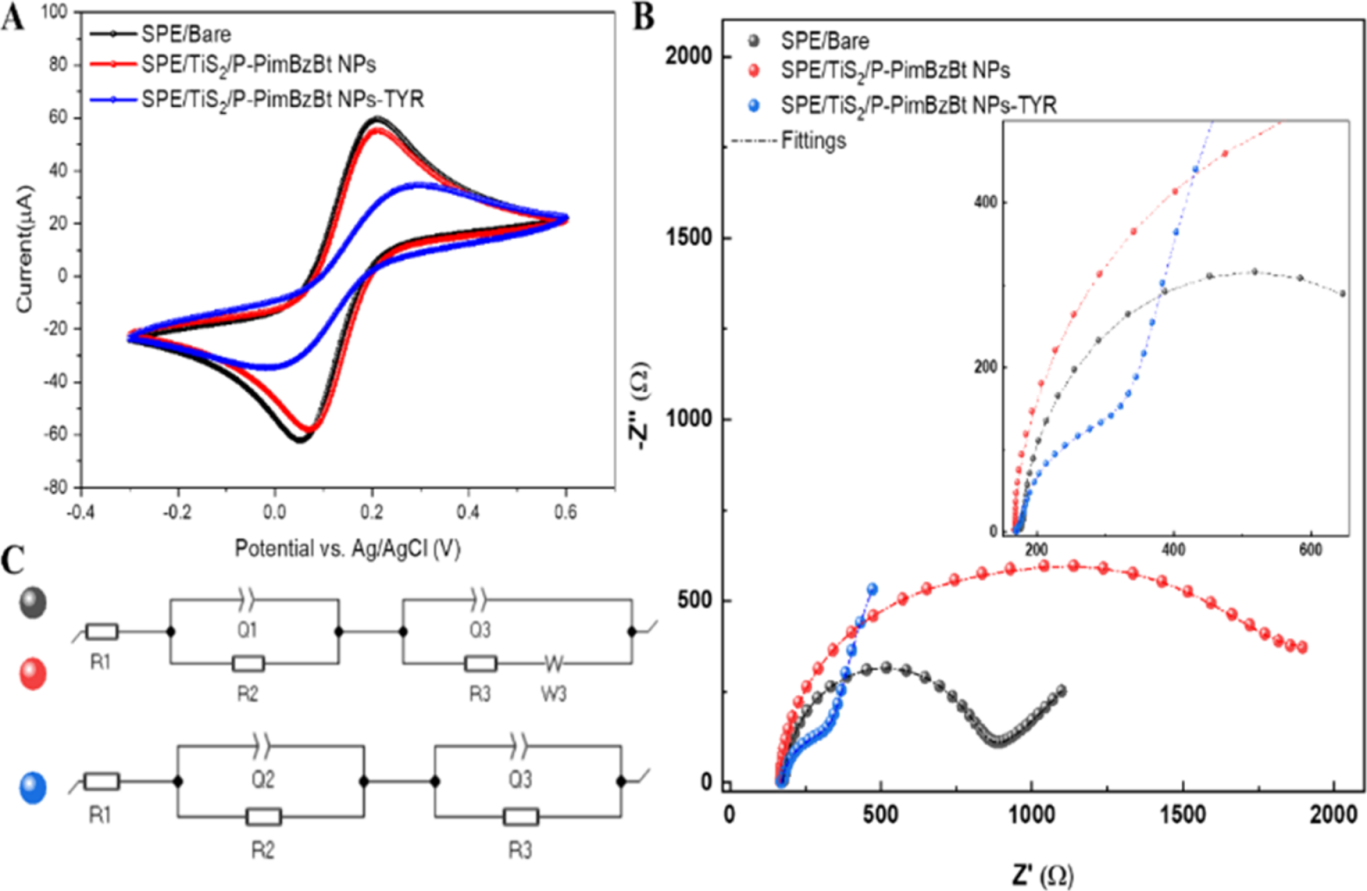
(A) CV and (B) EIS of bare SPE, SPE/TiS_2_/P-PimBzBt NPs, and SPE/TiS_2_/P-PimBzBt NPs/TYR in 50 mM buffer at pH 7.0 containing 5.0 mM [Fe(CN)_6_]^3–/4–^ and 0.1 M KCl. (C) Corresponding EIS circuit models.

The stepwise electrode modification process was confirmed by using electrochemical impedance spectroscopy (EIS) and cyclic voltammetry (CV) techniques. The CV responses of various modified electrodes are displayed in Figure 6A. The related experiments were performed in a 0.5 mM [Fe (CN)_6_]^3–/4–^ solution with 0.1 M KCl as the aqueous electrolyte at a scan rate of 50 mV/s. Using the Randles–Sevcik equation,^40^ the effective surface areas of the modified electrodes were determined to be 0.08 cm^2^, 0.07 cm^2^, and 0.043 cm^2^ for SPE/Bare, SPE/TiS_2_/P-PimBzBt NPs, and SPE/TiS_2_/P-PimBzBt NPs/TYR, respectively. The effective surface areas of TiS_2_/P-PimBzBt NPs/TYR were lower than those of the others because of insulator characteristics of the biomolecules that are commonly observed phenomena for enzyme-based electrodes.^41^ Another effective method for revealing details about the electrodes’ charge transport mechanism at the electrode–electrolyte interface is EIS. Using 0.1 M KCl with 5 mM [Fe(CN)_6_]^3–/4–^ as a redox probe in the frequency range of 1.0 Hz to 200 kHz, Nyquist plots of SPE/Bare, SPE/TiS_2_/P-PimBzBt NPs, and SPE/TiS_2_/P-PimBzBt NPs/TYR were examined (Figure 6B). Compared to the bare electrodes, TiS_2_/P-PimBzBt NPs/TYR showed a decreased surface area, contributing to a decrease in conductivity and an increase in *R*_ct_ values. Respective EIS models are given in Figure 6C to understand the effect of the addition of TYR to the system. SPE/Bare, SPE/TiS_2_/P-PimBzBt NPs, and SPE/TiS_2_/P-PimBzBt NPs/TYR all have an initial constant phase element (CPE) *Q*_2_, which can be correlated to the electrode–electrolyte interface. The second circuit containing *Q*_3_ contains information about the sensing capabilities of the samples. In all cases, the α constant of *Q*_3_ has values ∼0.98, indicating a strong capacitive behavior. On the other hand, *R*_ct_ values, which are indicated with *R*_3_, differ with each combination. The *R*_3_ values are found to be 636.1, 569.1, and 2928 Ω for SPE/Bare, SPE/TiS_2_/P-PimBzBt NPs, and SPE/TiS_2_/P-PimBzBt NPs/TYR, respectively. This is in correlation with the CV given in Figure 6A and supports the claim of the decreased surface area and decrease in conductivity. The lack of the Warburg element (*W*_3_) in SPE/TiS_2_/P-PimBzBt NPs/TYR can be explained by the prevention of diffusion at lower frequencies (1 Hz) due to the slower dielectric relaxation with the addition of TYR. All in all, the electrode modifications were successful, as evidenced by the CV and EIS data, which also showed strong agreement with one another.

**Figure 7:**
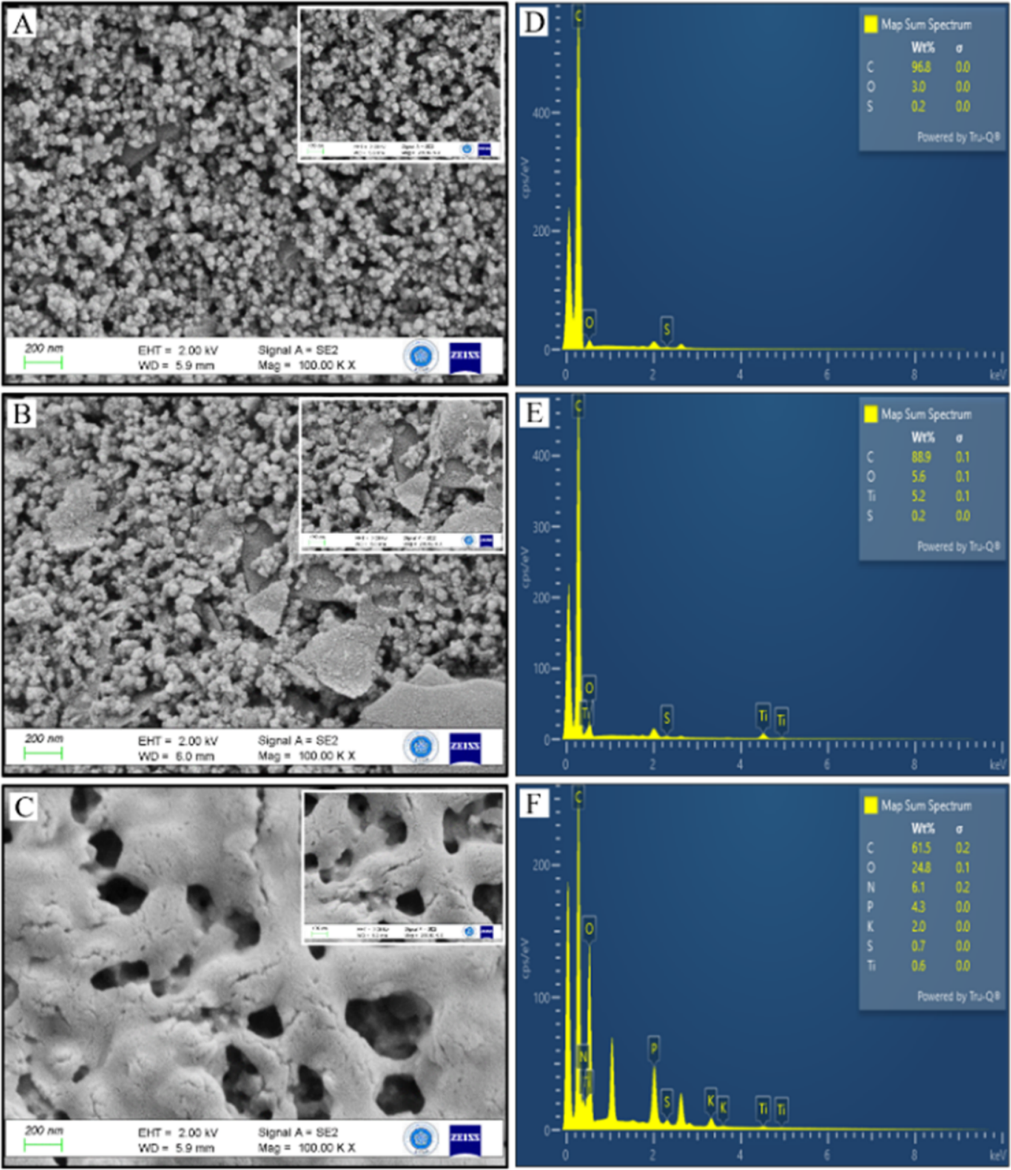
(A–C) FE-SEM and (D–F) EDS of SPE/P-PimBzBt NP, SPE/TiS_2_@P-PimBzBt NP, and SPE/TiS_2_@P-PimBzBt NPs/TYR samples.

FE-SEM and EDS analyses of SPE/P-PimBzBt NPs, SPE/TiS_2_/P-PimBzBt NPs, and SPE/TiS_2_/P-PimBzBt NPs/TYR are presented in Figure 7. The surface of SPE/P-PimBzBt NPs shows cauliflower-like structures as typical conjugated polymers, as seen in Figure 7A. P-PimBzBt forms nanoparticles in water with a size of about 90 nm, although the nanoparticles slightly adhere to each other instead of dispersing well. After TiS_2_ integration on the P-PimBzBt NP surface, Figure 7B reveals both morphologies of the structures on the electrode surface. The surface morphology of SPE/TiS_2_/P-PimBzBt NPs greatly changed after TYR deposition (Figure 7C). After each modification, all electrodes exhibit a homogeneous distribution of the materials. Elemental compositions of the samples on the prepared electrodes were given with EDS results (Figure 7D–F). Especially, the existence of the Ti element on the TiS_2_/P-PimBzBt NP- and SPE/TiS_2_/P-PimBzBt NPs/TYR-modified electrodes was proven, as shown in Figure 7E,F.

### 3.3 Optimization Studies of the Nanobiosensor

**Figure 8:**
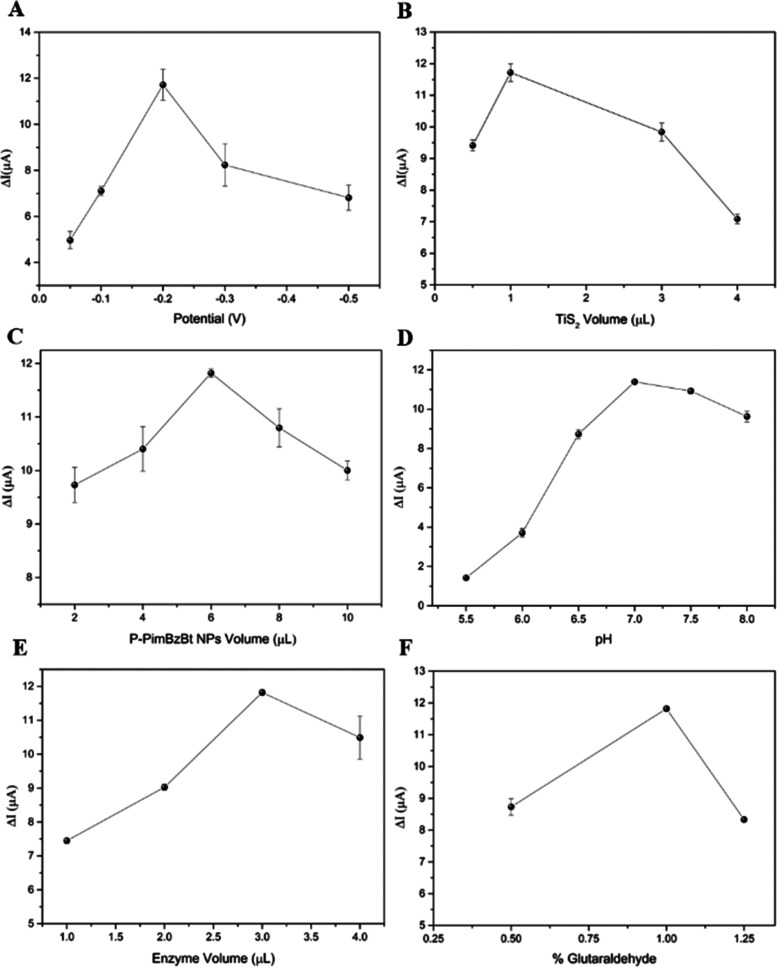
Optimization of the (A) voltage, (B) volume of TiS_2_, (C) P-PimBzBt NPs and (D) TYR, (E) percentage of GA, and (F) pH for the SPE/TiS_2_/P-PimBzBt NPs/TYR biosensor.

The proposed SPE/TiS_2_@P-PimBzBt NPs/TYR nanobiosensor was optimized using chronoamperometric responses for 25 μM catechol. First of all, the potential value of the working conditions was evaluated, and the ideal response was attained at a voltage of −200 mV (Figure 8A). Next, the amounts of P-PimBzBt NPs and TiS_2_ nanosheets were optimized. The best result was obtained with 1.0 μL of a droplet of TiS_2_ and 6.0 μL of P-PimBzBt NPs, respectively (Figure 8B,C). The pH effect was further investigated from pH 5.5 to 8.0, and optimum results were obtained with pH 7.0, as shown in Figure 8D. Later, the TYR amount was varied between 1.0 and 4.0 μL (Figure 8E). The ideal enzyme amount was found to be 3.0 μL. Finally, the percentage of glutaraldehyde utilized as a cross-linking agent was optimized. It was determined from the studies that the best outcome was obtained when 1.0% glutaraldehyde was used (Figure 8F).

### 3.4 Analytical Characterization of the SPE/TiS_2_/P-PimBzBt NPs/TYR Biosensor

**Figure 9:**
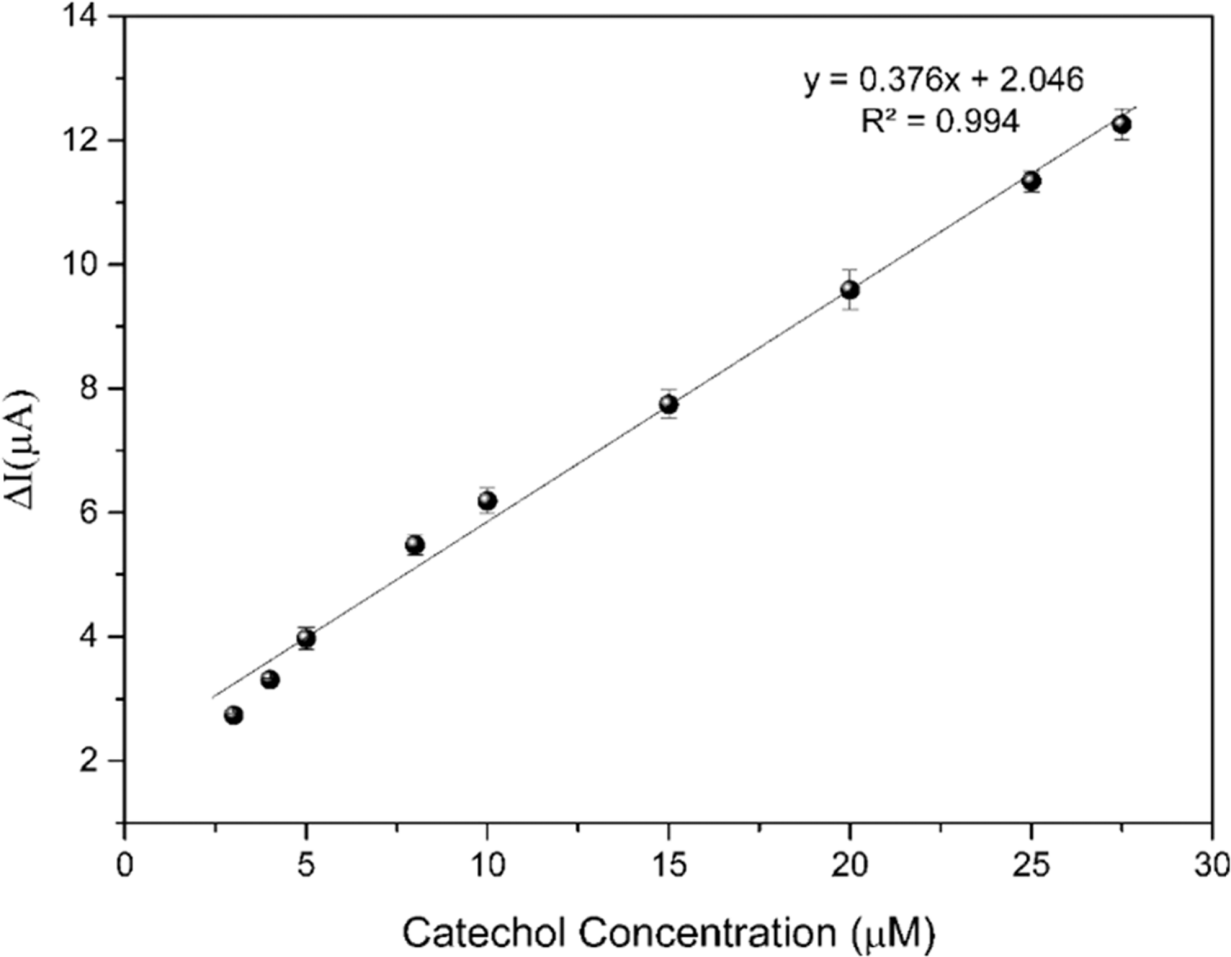
Amperometric current–time response curves of SPE/TiS_2_/P-BzPimBzBt NPs/TYR with successive additions of catechol with different concentrations into the reaction medium (50 mM pH 7.0 PBS; applied potential: −200 mV).

Analytical performances of SPE/TiS_2_@P-PimBzBt NPs/TYR were evaluated toward catechol after optimization studies. The analytical calibration curve for the electrochemical determination of catechol by amperometric measurements was created. Figure 9 displays the typical current–time curve of the present biosensor on the successive additions of various concentrations of catechol in PBS (pH 7.0) at an applied potential of −200 mV. When catechol was added, the steady-state current quickly altered and achieved a different steady-state current. These findings imply that the diffusion of catechol, *o*-quinone, H^+^, and O_2_ surrounding adsorbed TYR on the SPE/TiS_2_@P-PimBzBt NPs is reasonably smooth, which contributes to a rapid response for catechol. The current nanobiosensor displayed a linear range of catechol from 3.0 to 27.5 μM and the regression equation *y* = 0.376*x* + 2.046, with a coefficient of 0.994. The limit of detection (LOD) was estimated by setting the intercept of the linear range of the calibration curve to zero using S/N (signal-to-noise ratio) = 3 criteria. The LOD value was determined to be 0.33 μM, and the sensitivity was determined to be 3.89 μA/μM·cm^2^. The performance of the described catechol biosensors in comparison to that of the current SPE/TiS_2_@P-PimBzBt NPs/TYR biosensors is summarized in Table 1. In addition, Table 2 compares earlier biosensor research that utilizes TiS_2_ structures.

**Table 1:** Comparison of the Electrode Performance for Catechol Detection Using Differently Designed Electrodes^a^

name of the electrodes	target	mode	linear range (μM)	LOD (μM)	sensitivity (μA/μM·cm^2^)	application	refs
(Poly(RA)MGPE)	CT	DPV	2–10, 150–50	0.820, 2.760	NR	water	(42)
laccase/cysteine/gold SPE	CT	AMP	20–2400	1.2	3.93	NR	(43)
ALR/GCE	CT	DPV	0–80	0.126	NR	tap water	(44)
PRFMCNTPE	CT	CV, DPV	6–60, 0.5–7	0.025, 0.0039	NR	tap water	(45)
PGBAMCPE	CT	DPV	2–10	0.57	NR	black tea and tap water	(39)
Mg–Al LDHs/GCE	CT	DPV	0.007–200	0.0023	3.57	river water	(46)
TYR/AO/MLN/GCE	CT	AMP	0.1–80	0.029	0.0315	NR	(47)
Fc/APTMS/GO/GCE	CT	AMP	3–112	1.1	1.1843	lake water and tap water	(48)
Ag_2_O/Ag@NS-CQD/GCE	CT	AMP	0.2–180	0.013	0.7459	tap water and wastewater	(49)
PEDOT:PSS/20% IL/SPCE	CT	AMP	0.1–330	23.7	0.0182	river water and tap water	(50)
Po-DG6-MCPE	CT	AMP	20–160	0.09	NR	tap water	(51)
LAC/Cys-Ag@Fe_3_O_4_/CHIT/SPE	CT	AMP	0.1–100	0.06	0.060	lake water and tap water	(52)
SPE/TiS_2_@P-PimBzBt NPs/TYR	CT	AMP	3.0–27.5	0.33	3.89	black tea and tap water	TW

a CT: catechol, AA: ascorbic acid, EPC: epicatechin, UA: uric acid, IFN-γ: interferon-γ, AMP: chronoamperometry, DPV: differential pulse voltammetry, FL: fluorogenic, Poly(RA)MGPE: poly(rosaniline)-modified graphene paste electrode, SPGE: screen-printed gold electrode, ALR/GCE: Allura Red AC/glassy carbon electrode, PRFMCNTPE: electro-polymerized riboflavin-modified carbon nanotube paste electrode, CNTPE: carbon nanotube paste electrode, PGBAMCPE: poly gibberellic acid-modified carbon paste electrode, Co_3_O_4_/CSs/GCE: Co_3_O_4_/CSs-modified glassy carbon electrode, Mg–Al LDHs/GCE: magnesium–aluminum-layered double hydroxide/glassy carbon electrode, TYR/AO/MLNGCE: tyrosinase/acridine orange/natural molybdenite/glassy carbon electrode, Fc/APTMS/GO/GCE: ferrocene/(3-aminopropyl)trimethoxysilane/graphene oxide/glassy carbon electrode, Ag_2_O/Ag@NS-CQD/GCE: Ag_2_O, silver nanoparticles, and N,S-doped carbon quantum dot/glassy carbon electrode, PEDOT:PSS/20% IL/SPCE: poly(3,4-ethylenedioxythiophene):poly(4-styrenesulfonate)/ionic liquid/screen-printed carbon electrode, Po-DG6-MCPE: poly-DG6-modified carbon paste electrode, and LAC/Cys-Ag@Fe_3_O_4_/CHIT/SPE: laccase/l-cysteine-Ag@Fe_3_O_4_/chitosan/screen-printed electrode.

**Table 2:** Comparison of the Electrode Performance for Different Molecule Detection Using Electrodes Made with TiS_2_^a^

name of the electrodes	target	mode	linear range (μM)	LOD (μM)	sensitivity (μA/μM·cm^2^)	application	refs
erGO/TiS_2_/GCE	AA	AMP	0.1–1, 1–400	0.0302, 0.0914	0.34, 0.11	Limcee vit-C and urine	(53)
Ti_0.95_Nb_0.05_S_2_/GOx/GTA	GL	AMP	74.6–272.9, 0.767–12.6, 17.5–27.3	25.7	0.0179	human serum	(54)
LAC-GO/TiS_2_/NAF/SPE	EPC	AMP	0.1–250	0.073	0.3264	red wine fruit juice	(26)
TiS_2_ NSs	IFN-γ	FL	(0–3) × 10^–4^	82.7 × 10^–6^	NR	NR	(55)
Nafion/uricase/TiS_2_–AuNPs/SPE	UA	AMP	5–2000	0.18	0.2208	human serum	(56)
SPE/TiS_2_@P-PimBzBt NPs/TYR	CT	AMP	3.0–27.5	0.33	3.89	black tea and tap water	TW

a erGO/TiS_2_/GCE: electrochemically reduced graphen oxide/titanium disulfide/glassy carbon electrode, LAC-GO/TiS_2_/NAF/SPE: laccase-graphene oxide/titanium disulfate/Nafion/screen-printed electrode, and TiS_2_ NSs: titanium disulfate nanostructures.

**Figure 10:**
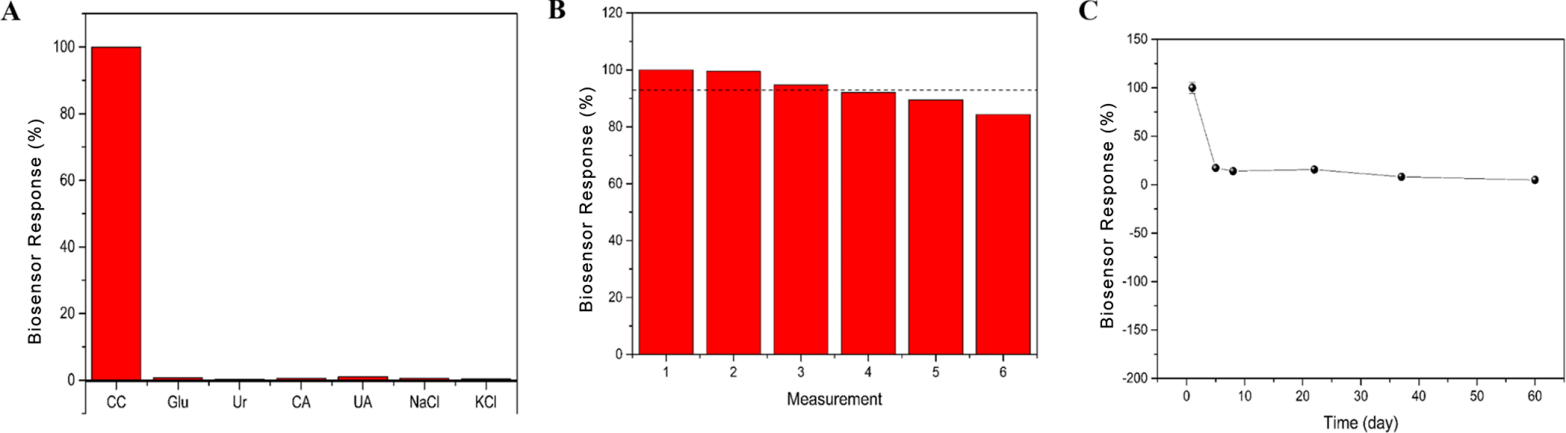
(A) Interference, (B) repeatability, and (C) shelf life results of the (SPE/TiS_2_)/P-PimBzBt NPs/TYR biosensor (the black dashed line indicates *I*_average_ of the results).

Ten successive amperometric responses of 25 μM catechol in 50 mM pH 7.0 phosphate buffer were used to examine the repeatability of the SPE/TiS_2_@P-PimBzBt NPs/TYR nanobiosensor. The SPE/TiS_2_@P-PimBzBt NPs/TYR nanobiosensor’s shelf life was assessed by keeping the same nanobiosensors at 4 °C for a continuous 37 days. The biosensors retained 15.6% of their activity at the end of the 22nd day (Figure 10). Additionally, the biosensing system was supplemented with commonly interfering ions and compounds (15 μM each) such as citric acid, urea, glucose, uric acid, KCl, and NaCl in order to track their interference on the SPE/TiS_2_@P-PimBzBt NPs/TYR nanobiosensor toward 15 μM catechol. Catechol sensing can be accomplished without any interference when these often-interacting ions and substances are present (Figure 10).

### 3.5 Sample Application

The SPE/TiS_2_@P-PimBzBt NPs/TYR nanobiosensor was utilized to measure the concentration of catechol in tap water and black tea samples. Measurements were performed using the standard addition procedure by spiking catechol of different concentrations into tap water and black tea samples. The reaction medium contains 10 mL of the working buffer, and different volumes of the catechol-containing samples were injected into the reaction medium. From the equation of the calibration curve, the catechol contents of these samples were determined. All of these experiments were performed under the optimum conditions and carried out at room temperature. For each concentration, three trial measurements were carried out for both the samples. We achieved a good recovery percentage of over 95%, as shown in Table 3. This authenticates the ability of the SPE/TiS_2_@P-PimBzBt NPs/TYR nanobiosensor for determining catechol in real samples.

**Table 3:** Results of the SPE/TiS_2_@P-PimBzBt NPs/TYR Biosensor for Real-Time Applications

sample	spiked with catechol (μmol/L)	found with the biosensor (μmol/L)	recovery (%)
tap water	15	14.33 ± 0.20	95.55
	25	25.06 ± 1.27	100.27
black tea	5	4.92 ± 0.87	98.39
	10	10.08 ± 0.16	100.84
	15	16.00 ± 0.07	106.72

### 3.6 Computational Results

MD simulations showed that the interaction energy of P-BzPimBzBt with graphene is lower than that with graphene/TiS_2_ calculated as −225 and −371 kcal/mol, respectively, for the tetramer at the interface. The increased interaction energy indicated the contribution of the TiS_2_ layer to the stability of the nanobiosensor. The increased interaction energy by the presence of TiS_2_ was monitored by the decrease in the mobility of the chains at the interface given in SI-movie1 and SI-movie2 for graphene@P-PimBzBt and graphene/TiS_2_@P-PimBzBt, respectively. While P-PimBzBt has only a π–π interaction with the nonpolar graphene surface, graphene/TiS_2_ can create dipole–dipole interactions with the chains due to the partial negative charges on the S atom of the TiS_2_ layer calculated as −0.45 e on average by the Qeq method.

More accurate single-point DFT calculations show a similar charge distribution by the electrostatic potential charge algorithm.^57^ Here, partial negative charges on the S atom of the TiS_2_ layer were calculated as −0.51 e for the single polymer repeat unit on the surface. These extra nonbonding interactions provided by the TiS_2_ layer were reflected to the total interaction energies calculated as −47 kcal/mol for graphene and −113 kcal/mol for graphene/TiS_2_. Charge transfers were calculated as −0.11 e for graphene to P-PimBzBt and −0.76 e for graphene/TiS_2_ to P-PimBzBt. This means that the graphene/TiS_2_ complex not only increased the stability of the nanobiosensor but also increased the charge transfer between layers, which is a desired property for sensing. Another difference between the structural properties of the systems is the position and configuration of the P-PimBzBt repeat unit on the surfaces. Aromatic centers of the P-PimBzBt repeat unit were positioned planarly on the top of graphene carbons for the graphene@P-PimBzBt systems. However, carbon and hydrogen atoms of the P-PimBzBt repeat unit with partial positive charges were positioned on top of the S atoms of TiS_2_ for the graphene/TiS_2_@P-PimBzBt system. We concluded that graphene/TiS_2_@P-PimBzBt can form a stable nanostructure and nanobiosensor properties due to the conductivity of the P-PimBzBt chains and the increased interaction with the polymer layer by the addition of the TiS_2_ layer between graphene and P-PimBzBt, as well as the increased polarity and charge transfer provided by the TiS_2_ layer.

## 4 Conclusions

This study shows the use of novel TiS_2_@P-PimBzBt NP composites as electrochemical biosensors for the identification of catechol. With this work, a novel polymer and its nanoparticles were synthesized and characterized, and its composite with 2D-TiS_2_ nanosheets was produced for the first time. With the following benefits, these TiS_2_@P-PimBzBt NP composites are promising materials for catechol biosensors that detect interference-free catechol in real samples. The TiS_2_ and P-PimBzBt NP combination provided improved sensing ability, and the importance of the related composition was confirmed by DFT calculations. Theoretical studies presented that the TiS_2_ layer not only leads to the improved self-organization of the chains and interfacial interactions but also alters the electronic properties through charge transfer. The related combination not only improved the electrochemical properties of the system but also served as a great microenvironment for enzyme immobilization platforms for sensing experiments. The developed biosensor, which has a linear range of 3.0–27.5 μM and a detection limit of 0.33 μM, offers significant sensitivity and specificity for the detection of catechol. The biosensor makes it possible to accurately determine the quantities of catechol, even in black tea and tap water samples.

## Supporting Information Available

DLS and ζ potential results of the P-PimBzBt NPs (PDF)
Molecular dynamics simulation trajectory for the P-PimBzBt on the graphene surface (ZIP)
Molecular dynamics simulation trajectory for the P-PimBzBt on the graphene/TiS_2_ surface (ZIP)

## Abbreviations

TYR: tyrosinase
2D-TiS_2_: two-dimensional titanium disulfide nanosheets
CV: cyclic voltammetry
EIS: electrochemical impedance spectroscopy
FE-SEM: field emission scanning electron microscopy
EDS: energy-dispersive X-ray spectroscopy
CPs: conjugated polymers
CPNs: conjugated polymer nanoparticles
TiS_2_: titanium disulfide
TMDs: transition metal dichalcogenides
P3HT: poly(3-hexylthiophene)
THF: tetrahydrofuran
DLS: dynamic light scattering
MD: molecular dynamics
NMR: nuclear magnetic resonance
GPC: gel permeation chromatography
CPE: constant phase element
*W*: Warburg element
LOD: limit of detection
