# Supplementary material for: Two-Dimensional TiS2 Nanosheet- and Conjugated Polymer Nanoparticle-Based Composites for Sensing Applications
Source: Langmuir. 2024 Oct 15;40(43):22960–72. doi: 10.1021/acs.langmuir.4c03102 (PMC11526354; doi:10.1021/acs.langmuir.4c03102)
Supplement: Supplementary file 1 — la4c03102_si_001.pdf [file la4c03102_si_001.pdf]

Supporting Information

Two-Dimensional TiS2 Nanosheet and Conjugated Polymer Nanoparticles- Based Composites for Sensing Applications

D.Yeniterzi[†, ‡], S.C.Cevher[§], S.Kandur[¥], A.D.Ucar[¥], M.B.Durukan[¥], T.Haciefendioglu[§], E.Yildirim[§], A. Cirpan[§]*, H.E.Unalan[¥, ¤,**], S.Soylemez[†, ‡,***]

†Department of Biomedical Engineering, Necmettin Erbakan University, 42090, Konya, Turkey

‡Science and Technology Research and Application Center (BİTAM), Necmettin Erbakan University, 42090, Konya, Turkey

§Department of Chemistry, Middle East Technical University, Ankara 06800, Turkey

¥Department of Metallurgical and Materials Engineering, Middle East Technical University (METU) 06800 Ankara, Turkey

¤Energy Storage Materials and Devices Research Center (ENDAM), Middle East Technical University (METU) 06800 Ankara, Turkey

Table of Contents

Figure S1.DLS Analysis Results of P-PimBzBt Conjugated Polymer Nanoparticles............................S-2
Figure S2.Zeta Potential Results of P-PimBzBt Conjugated Polymer Nanoparticles............................S-3

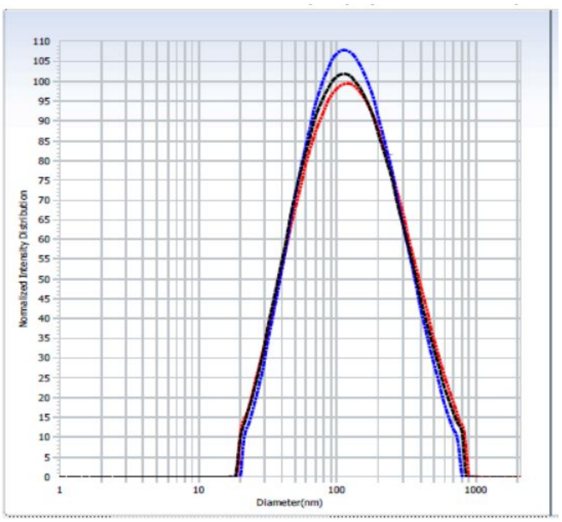

Measurement Number	Hydrodynamic Diameter averages (nm)	PDI (Poly Dispersty Index)
1	91.1	0.348
2	95.1	0.329
3	95.9	0.346
**Avarege**	**94.0**	**0.341**

Figure S1.DLS results of the P-PimBzBt conjugated polymer nanoparticles

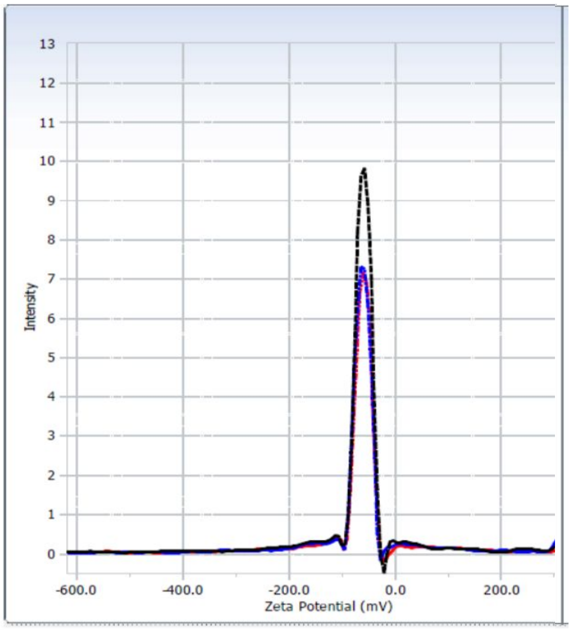

Measurement Number	Zeta Potential (mV)
1	-60.04
2	-61.71
3	-59.79
**Average**	**-60.52**

Figure S2. Zeta potential results of the P-PimBzBt conjugated polymer nanoparticles
