# Supplementary material for: Two-Dimensional TiS2 Nanosheet- and Conjugated Polymer Nanoparticle-Based Composites for Sensing Applications
Source: Langmuir. 2024 Oct 15;40(43):22960–72. doi: 10.1021/acs.langmuir.4c03102 (PMC11526354; doi:10.1021/acs.langmuir.4c03102)
Supplement: Supplementary file 2 — la4c03102_si_002.zip [file la4c03102_si_002.zip › SI-Movie1.pptx]

## Slide 1
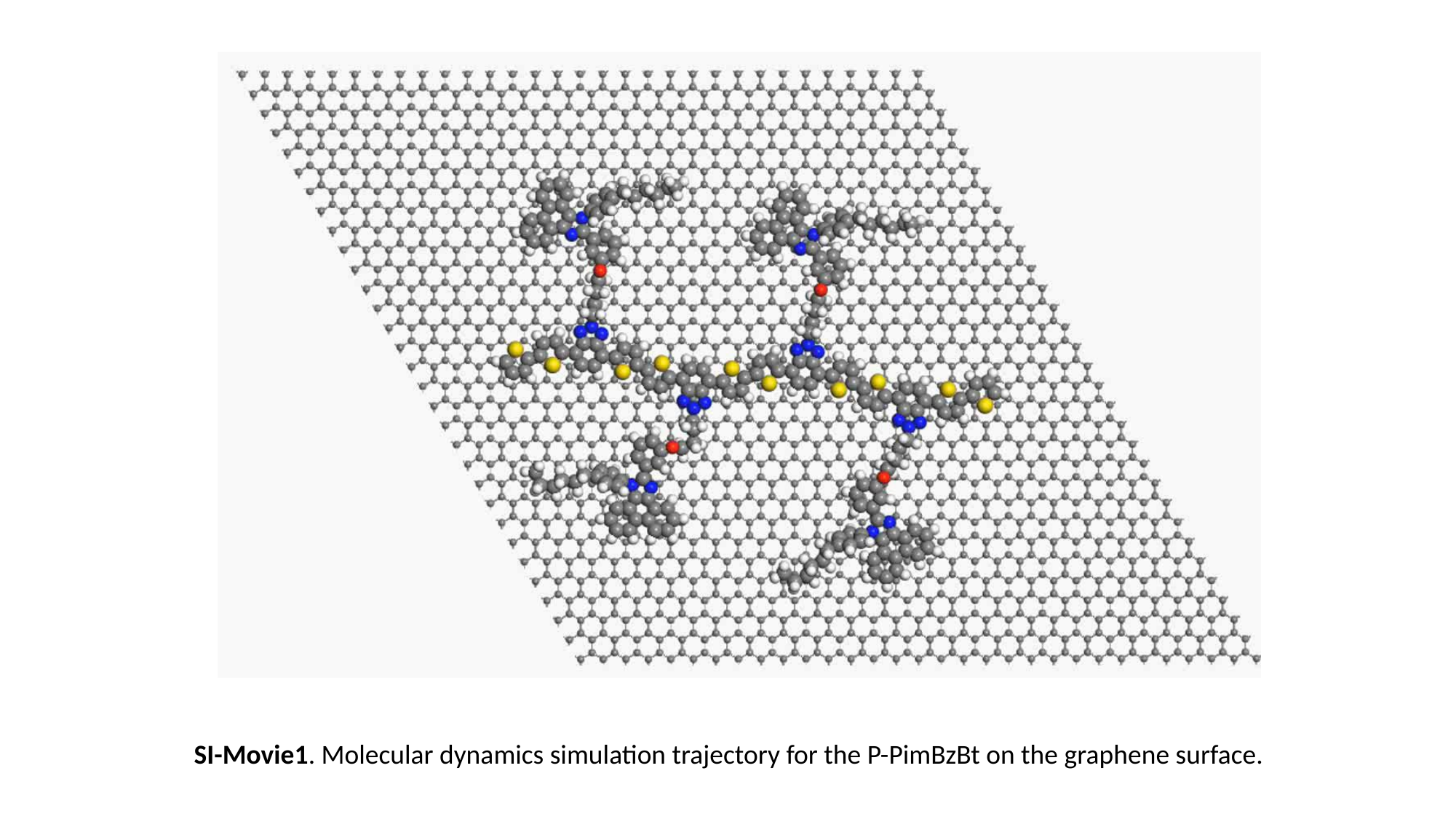

SI-Movie1. Molecular dynamics simulation trajectory for the P-PimBzBt on the graphene surface.
